# Optimizing antiviral treatment for seasonal influenza in the USA: a mathematical modeling analysis

**DOI:** 10.1186/s12916-021-01926-5

**Published:** 2021-03-01

**Authors:** Matan Yechezkel, Martial L. Ndeffo Mbah, Dan Yamin

**Affiliations:** 1 Department of Industrial Engineering, Tel Aviv University, 55 Haim Levanon St, Tel Aviv, Israel; 2 Department of Veterinary Integrative Biosciences, College of Veterinary Medicine & Biomedical Sciences, Texas A&M University, College Station, Texas 77843 USA; 3 Department of Epidemiology and Biostatistics, School of Public Health, Texas A&M University, Texas, 77843 USA; 4 Center for Combatting Pandemic, sTel Aviv University, 55 Haim Levanon St, Tel Aviv, Israel

**Keywords:** Seasonal influenza, Antiviral treatment, Vaccination, Mathematical modeling

## Abstract

**Background:**

Seasonal influenza remains a major cause of morbidity and mortality in the USA. Despite the US Centers for Disease Control and Prevention recommendation promoting the early antiviral treatment of high-risk patients, treatment coverage remains low.

**Methods:**

To evaluate the population-level impact of increasing antiviral treatment timeliness and coverage among high-risk patients in the USA, we developed an influenza transmission model that incorporates data on infectious viral load, social contact, and healthcare-seeking behavior. We modeled the reduction in transmissibility in treated individuals based on their reduced daily viral load. The reduction in hospitalizations following treatment was based on estimates from clinical trials. We calibrated the model to weekly influenza data from Texas, California, Connecticut, and Virginia between 2014 and 2019. We considered in the baseline scenario that 2.7–4.8% are treated within 48 h of symptom onset while an additional 7.3–12.8% are treated after 48 h of symptom onset. We evaluated the impact of improving the timeliness and uptake of antiviral treatment on influenza cases and hospitalizations.

**Results:**

Model projections suggest that treating high-risk individuals as early as 48 h after symptom onset while maintaining the current treatment coverage level would avert 2.9–4.5% of all symptomatic cases and 5.5–7.1% of all hospitalizations. Geographic variability in the effectiveness of earlier treatment arises primarily from variabilities in vaccination coverage and population demographics. Regardless of these variabilities, we found that when 20% of the high-risk individuals were treated within 48 h, the reduction in hospitalizations doubled. We found that treatment of the elderly population (> 65 years old) had the highest impact on reducing hospitalizations, whereas treating high-risk individuals aged 5–19 years old had the highest impact on reducing transmission. Furthermore, the population-level benefit per treated individual is enhanced under conditions of high vaccination coverage and a low attack rate during an influenza season.

**Conclusions:**

Increased timeliness and coverage of antiviral treatment among high-risk patients have the potential to substantially reduce the burden of seasonal influenza in the USA, regardless of influenza vaccination coverage and the severity of the influenza season.

**Supplementary Information:**

The online version contains supplementary material available at 10.1186/s12916-021-01926-5.

## Background

Seasonal influenza continues to impose major health and economic burdens [1, 2]. Although influenza is generally a self-limiting disease, it can result in severe illness and death. In particular, influenza imposes a substantial health burden on young children, the elderly population, and people with certain health conditions [3, 4]. In the USA, seasonal influenza results in an estimated 9.3–49.0 million illnesses, 140,000–710,000 hospitalizations, and 12,000–56,000 deaths annually [1, 5].

Vaccination is the mainstay of efforts to reduce the burden of seasonal influenza. The US Advisory Committee on Immunization Practices (ACIP) recommends influenza vaccination for all individuals aged six months or older. However, the majority of the US population does not comply with this recommendation, and rates of vaccination against seasonal influenza remain at approximately 40% annually [6]. Additionally, due to the rapid mutation of the virus and an imperfect match between the circulating strains and those covered by the vaccine, vaccine efficacy is not perfect and varies widely by season. For example, the average influenza vaccine effectiveness was estimated to be 45% in the USA, with the annual value ranging between 19 and 60% over the past decade [7, 8].

Recently, increased severity of influenza, marked by high outpatient and inpatient visits, has been observed among patients at high risk for influenza-associated complications [9, 10]. This group includes children under two years old, adults over 65 years old, people in certain racial-ethnic minority groups, pregnant women, and people with certain medical conditions [11]. This high-risk population accounts for the bulk of influenza-associated hospitalizations in the USA. For example, more than 50% of all influenza-associated hospitalizations in the USA occur among adults over 65 years old, and more than 70% of adult inpatients have at least one underlying medical condition that places them at high risk for influenza-associated complications [9, 12–14].

The increases in influenza-associated hospitalization and mortality [10, 15] have raised concerns about vaccine uptake and the early treatment of patients at risk of severe complications [16, 17]. Neuraminidase inhibitors (NAIs) are a class of antiviral medications recommended for the pharmacologic treatment of influenza [18]. Early treatment of influenza patients with NAIs reduces the duration and intensity of viral shedding, the duration of symptoms, and disease-associated complications, hospitalizations, and mortality [19–22]. Despite the high burden of influenza-induced complications among high-risk individuals, their rate of treatment for influenza has remained low [23]. Approximately 40% of high-risk patients with laboratory-confirmed influenza seek care within 2 days of symptom onset [23]. Among these patients, on average, 37% are prescribed an antiviral medication [23, 24]. The ACIP guidelines recommend administering antiviral treatment to high-risk patients with clinically suspected influenza, even when laboratory confirmation is delayed, when influenza is known to be circulating in the population [18]. However, current clinical practice is far from adhering to these guidelines.

Antiviral treatment not only provides direct benefits to treated patients by reducing their risk of influenza-induced hospitalization and/or mortality but may also provide indirect protection to noninfected individuals by reducing their risk of infection. This indirect benefit is achieved by decreasing the contribution of treated patients to disease transmission by reducing their viral shedding and the duration of infectiousness. Household-based trials have shown that the early treatment of infected individuals with NAIs may reduce their contribution to disease transmission by 50–80% [25, 26].

Transmission models can be used to evaluate the indirect effect of interventions that arise from reduced transmission [27–31]. Specifically, models incorporating antiviral treatments showed that antiviral treatment in pandemic settings could avert substantial numbers of cases and hospitalizations [32, 33]. The logarithm of the infectious viral load is correlated with the transmissibility of several respiratory viruses, including influenza [34–36]. Thus, several transmission models that explicitly consider the daily viral load showed accurate projections of viral infections [34, 37–39]. Specifically, the unique viral load shedding pattern of influenza, which typically peaks around the day of symptom onset [40], suggests that antiviral treatment provided shortly after symptom onset can have a substantial effect on transmission.

To evaluate the population-level impacts of increased antiviral treatment coverage and timeliness of influenza treatment among high-risk individuals during influenza seasons in four states, namely, California, Connecticut, Texas, and Virginia, we developed a data-driven influenza transmission model that incorporates data on infectious viral load, social contact, healthcare-seeking behavior, time to seek healthcare, and antiviral treatment. Our model enables us to explicitly explore the effectiveness of antiviral treatment throughout the course of the disease. Our work emphasizes the importance of investing more effort by health officials to increase the early administration and coverage of antiviral treatment among infected high-risk individuals.

## Methods

### Model overview

**Fig. 1:**
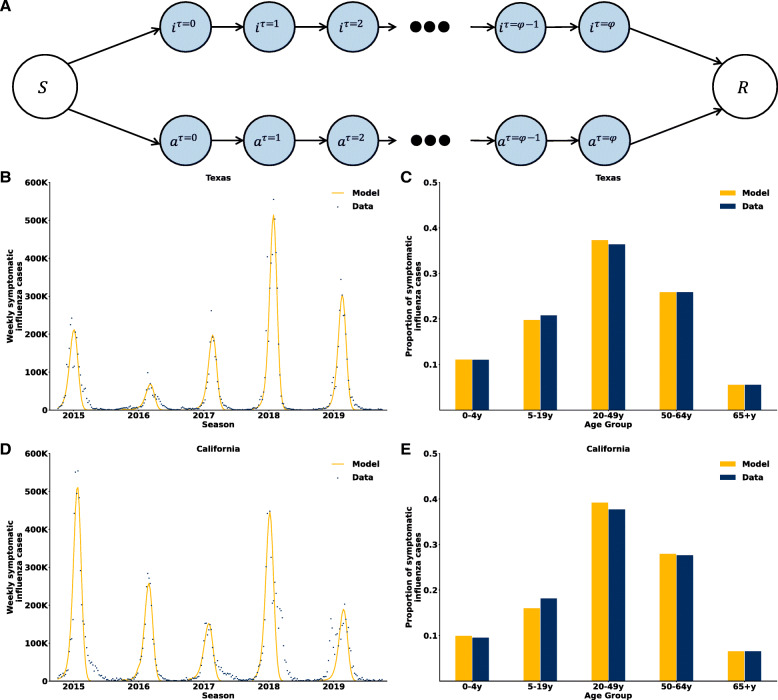
Structure and fit of the model. **a** Compartmental diagram of the transmission model. Susceptible individuals *S* move, following exposure, to the symptomatic or asymptomatic compartment, i^τ = 0^, a^τ = 0^, representing the first day of infection. The model explicitly tracks the day of infection τ such that the daily transmissibility is based on the daily viral load [41, 42]. Upon recovery, individuals transition to an *R* compartment, at which point they are fully protected for the remainder of the season. We also incorporated five age groups into the model, namely, 0–4, 5–19, 20–49, 50–64, > 65 years, and two risk groups within each age group, namely, high-risk and low-risk. Age and risk stratifications are not displayed to increase clarity in the diagram (Additional file 1). **b**, **d** Time series of recorded weekly symptomatic influenza cases and model fit to California and Texas (the model fit to Connecticut and Virginia is provided in Additional file 1*,* Figure S2). **c**, **e** Data and model fit to the age distribution among influenza cases

We developed a dynamic model of influenza infection progression and transmission in Texas, California, Connecticut, and Virginia. Our model is a modified susceptible-infected-recovered compartmental framework [27], where by the population is stratified into health-related compartments, and transitions between the compartments occur over time (Fig. 1a). Transitions between the compartments are governed by a series of difference equations (Additional file 1: Model overview, equations 2) [1, 5, 7, 8, 20, 23, 24, 27, 34–37, 41–76]. To model age-dependent transmission, we stratified the population into five age groups: 0–4 years, 5–19 years, 20–49 years, 50–64 years, and ≥ 65 years. We also distinguished between high-risk and low-risk individuals within each age group based on the ACIP case definition [18]. Altogether, our model includes (5 × 2 = 10) ten subgroups based on age and risk.

At the beginning of each season, susceptible individuals from age group *j* are in the *S*_*j*_ compartment. In the model, susceptible individuals may interact with infectious individuals and become either asymptomatically or symptomatically infected [72, 73], at which point they can transmit the disease to others until they recover. Upon infection, individuals transition into the *i*^*τ* = 0^, *a*^*τ* = 0^ compartments, representing the first day of their symptomatic or asymptomatic infection, respectively. Consistent with previous models [34, 37–39], we explicitly tracked the day of infection *τ* such that the daily transmissibility was based on the daily viral load [41, 42]. Upon recovery, individuals transition to a *R*_*j*_ compartment, at which point they are fully protected for the remainder of the season. This assumption is supported by prospective studies demonstrating that reinfection within the same season is rare [51, 52] (Fig. 1).

### Force of infection

The rate at which infectious individuals transmit the virus depends on (1) age-specific contact rates (Additional file 1: Table S1) between an infected individual and his or her contacts, (2) age-specific susceptibility to infection, and (3) infectiousness of the infected individual based on her/his daily viral loads and the time in the season (Additional file 1: Figure S1; Additional file 1 for details).

We parameterized the age-specific contact rates between an infected individual in age group *e* and their contact in age group *j*, *C*_*e*, *j*_ using data from an extensive survey of daily contacts (Additional file 1: Table S1) [34, 59]. These contact data exhibit frequent mixing between similar age groups, moderate mixing between children and adults in their thirties, and infrequent mixing between other groups. Age-specific susceptibility to infection *β*_*j*_ was calibrated using data on the weekly numbers of cases of influenza (see the “Model calibration” section).

The logarithm of the infectious viral load positively correlates with the transmissibility of several respiratory viruses, including influenza [34–37, 58]. The viral load depends on (1) the day of infection, (2) the risk group of the infected individual (i.e., high-risk/low-risk), (3) the type of infection (symptomatic/asymptomatic), and (4) the day on which the individual received antiviral treatment. We evaluated the viral load, detailed in Additional file 1: Datasets and parameters, by explicitly accounting for these four components based on recent prospective studies (using real-time RT-PCR) that observed young children and adults over the course of their influenza infection with and without treatment [41, 42]. These studies suggest that the viral load in infected individuals peaks on approximately the day of symptom onset. Moreover, untreated infected individuals at high risk exhibited the most viral shedding, while asymptomatic individuals had the least viral shedding. Based on the reduction in the viral shedding, we considered 23.2% (CI 10.4–34.3%) reduction in transmissibility for individuals at high risk who are treated within 0–48 h of symptom onset. Likewise, individuals treated within 48–72 from symptoms onset had 21.1% (CI 9.3–32.2%) [37, 41].

In the USA, influenza incidence is seasonal, with a peak typically occurring in the winter. However, the driver of this seasonality remains uncertain [53]. Thus, we included general seasonal variation in the susceptibility rate of the model as $T(t)=\left(1+\mathit{\cos}\left[\frac{2\pi \left(t-\phi \right)}{365}\right]\right)$, where *ϕ* is a seasonal offset. This formulation was previously shown to capture the seasonal variation in the incidence of respiratory diseases in the USA [34, 54, 77]. Altogether, the force of infection for age group *j*, *λ*_*j*_(*t*), is given by:

$$ {\lambda}_j(t)={\beta}_j\cdot T(t)\cdot \left(\sum \limits_{e=1}^5{C}_{e,j}\left(\sum \limits_{\tau =0}^{\varphi}\sum \limits_{k\in \left\{H,L\right\}}V{L}_{k,S}^{\tau}\cdot {i}_{e,k}^{\tau}\left(t-1\right)+\sum \limits_{\tau =0}^{\varphi}\sum \limits_{k\in \left\{H,L\right\}}V{L}_{k,A}^{\tau}\cdot {a}_{e,k}^{\tau}\left(t-1\right)\right)\right), $$

where *β*_*j*_ is the susceptibility rate for an individual in age group *j* and $V{L}_{k,S}^{\tau }$ is the logarithm of the viral load in symptomatic individuals on the day of infection *τ* in risk group *k* ∈ {*H*, *L*} (i.e., high- and low-risk). $V{L}_{k,A}^{\tau }$ is the logarithm of the viral load in asymptomatic individuals on the day of infection *τ* in risk group *k*. The numbers of symptomatic and asymptomatic infected individuals are represented by ${i}_{j,k}^{\tau },\kern0.5em {a}_{j,k}^{\tau }$. Likewise, we incorporated the effect of antiviral treatment. A detailed description is presented in Additional file 1: Force of Infection.

We chose Texas, California, Connecticut, and Virginia because they reflect the large variability in the USA in terms of population size, climatic factors, geographic location, sociodemographic characteristics, and vaccination coverage. Specifically, Texas and California are the most populated states, with 28,995,881 and 39,512,223 residents, respectively, while Virginia and Connecticut have smaller populations, with 8,535,519 and 3,565,287 residents, respectively [78]. The median ages of the residents of Texas and California are 35.0 and 37.0, respectively. In Virginia and Connecticut, the population is relatively older, with median ages of 38.6 and 41.1, respectively [79, 80]. The vaccination coverage levels in Virginia and Connecticut are above the national average, while those in California and Texas are below the national average [69]. Thus, these four states are arguably adequately representative of the USA.

### Hospitalizations

Hospitalization was not modeled explicitly. However, the number of hospitalizations in each age and risk group was computed by multiplying the number of symptomatic infected individuals by the rate of hospitalization given influenza infection (Table 1). These age- and risk-specific rates were obtained from epidemiological studies [60–62] (see Additional file 1: Fixed parameters).

**Table 1:** Model parameters

Parameter	Values in the baseline scenario	Ranges tested in the sensitivity analysis^**1**^	Justifications
**Percentage of individuals at high risk**			
Age group, years:			
0–4	5.0%		[1]
5–19	10.6%		
20–49	14.9%		
50–64	33.0%		
≥ 65	51.2%		
**Percentage of individuals at high risk who receive treatment within 48 h after symptom onset**			
Age group, years:			
0–4	4.3%	4.3–100.0%	[1, 23, 24]
5–19	2.7%	2.7–100.0%	
20–49	4.8%	4.8–100.0%	
50–64	3.7%	3.7–100.0%	
≥ 65	3.1%	3.1–100.0%	
**Percentage of individuals at high risk who receive treatment more than 48 h after symptom onset**			[24]
Age group, years:			
0–4	11.6%		
5–19	7.3%		
20–49	12.8%		
50–64	9.9%		
≥ 65	8.4%		
**Percentage reduction in transmissibility from individuals who are treated within 0–48 h**^**2**^	23.2%	10.4–34.3%	[37, 41]
**Percentage reduction in transmissibility from individuals who are treated within 48–72 h**^**2**^	21.1%	9.3–32.2%	[37, 41]
**Effectiveness of administering antiviral treatment within0–72 h in reducing hospitalizations**^**2**^			
≤ 19 years	75.0%	Uniform (11,81)	[20, 64–68]
> 19 years	59.0%	Uniform (11,89)	
**Case: hospitalization ratio**			
Age group, years:			
0–4	143.4:1		[81]
5–19	364.7:1		
20–49	178.2:1		
50–64	94.3:1		
≥ 65	11.0:1		
**High-risk: low-risk hospitalization ratio**			
Age group, years:			
0–4	10.7:1		[61, 62]
5–19	8.0:1		
20–49	8.0:1		
50–64	6.8:1		
≥ 65	3.0:1		
**Susceptibility rate**			
Age group, years:			
0–4	0.0026–0.0036		Additional file 1: Calibrated parameters
5–49	0.0014–0.0016		
50–64	0.0028–0.0032		
≥ 65	0.0020–0.0022		

^1^ Figures 2, 3, 4 and in uncertainty Table 3

^2^ Treatment provided following 72 h is considered to have no effect in reducing both hospitalizations and transmission

High-risk individuals receiving antiviral treatment have a lower rate of hospitalization than nontreated high-risk patients. The age-specific reduction in hospitalization rate among treated high-risk individuals was obtained from previous retrospective and prospective studies (Table 1) [64, 65].

### Baseline vaccination and treatment

For each year, we parameterized vaccination coverage from state-specific influenza vaccine coverage data for different age groups, as observed from 2013 to 2018 (Additional file 1: Table S2) [69]. We estimated vaccine efficacy using the Centers for Disease Control and Prevention (CDC) estimates for influenza vaccine efficacy between 2013 and 2018 [7].

The proportion of individuals at high risk receiving antiviral drugs and the timing of antiviral administration after symptom onset were obtained from large-scale studies among US patients [24] (Table 1; Additional file 1: Fixed parameters). These data were used to inform our baseline treatment scenario for each state.

### Model calibration

To empirically estimate unknown epidemiological parameters, we calibrated our model to the data on the weekly numbers of cases of influenza (confirmed by viral isolation, antigen detection, or PCR) [75]. These data were collected by the National Respiratory and Enteric Virus Surveillance System of the CDC and state health departments from four different states in the USA from 2014 to 2019.

To obtain the numbers of influenza cases, we multiplied the number of weekly influenza-like illness (ILI) cases by the weekly proportion of specimens positive for influenza. To account for the unreported cases in each age group, we scaled up the number of cases such that the mean attack rate between 2014 and 2019 matched large-scale estimates from a meta-analysis conducted in the USA [5]. Although several studies have attempted to estimate state-level annual influenza attack rates in the USA [1, 5, 76], the state-level rates remain unknown. Therefore, we used the national attack rate to scale up state-specific numbers of influenza cases. Altogether, the yearly attack rate varied considerably between years and states, ranging from 2.8–15.0% in Texas, 4.5–12.0% in California, 5–11.7% in Connecticut, and 4.0–12.3% in Virginia.

Due to the uncertainty related to the actual incidence of influenza, we calibrated our model parameters for each state using different settings reflecting the lowest, average, and highest attack rates across influenza seasons.

### Interventions

We evaluated two interventions for increasing the number of high-risk patients seeking care and being treated within the first 2 days. In the first intervention, we increased the number of individuals who were treated within the first two days after symptom onset by assuming that a proportion of those who received treatment after this infection period would receive treatment within the first two days after symptom onset. In the second intervention, we increased the total number of infected high-risk individuals who received treatment while assuming that they all received treatment within the first 2 days after symptom onset. We evaluated the population- and individual-level benefits of these interventions in terms of infections and hospitalizations averted during a single influenza season.

### Sensitivity analyses

We conducted sensitivity analyses to examine the robustness of our results. In the first analysis, we investigated the impact of effective vaccination coverage on the effectiveness of antiviral treatment. Effective vaccination coverage is defined as the product of vaccine efficacy times vaccine coverage and represents the level of vaccine-induced immunity in the population. In the second analysis, we conducted a two-way sensitivity analysis to investigate the joint impact of changing both the attack rate and the effective vaccination coverage. There is a high variability in the literature concerning the effectiveness of early antiviral treatment in reducing hospitalizations [20, 64–68]. Thus, we also conducted a sensitivity analysis that considered two main aspects: (1) changes in transmissibility that arise from uncertainty related to the effect of the reduction in the viral load following treatment and (2) uncertainty of the effectiveness of treatment with regard to preventing hospitalizations. Uncertainty regarding the daily reduction in viral load was based on estimates from a clinical trial [42]. We considered the effectiveness of early treatment to range between 11 and 89% in adults and 11 and 81% in children (Table 1).

### Model simulations

To determine the population-level and the per person treated benefit of increasing the coverage and timeliness of antiviral treatment, we simulated five influenza seasons with the implementation of the interventions. We evaluated the cases and hospitalizations averted for a range of values of effective vaccination coverage, attack rate, and early treatment coverage. We define early treatment if antiviral treatment is administered within 48 h. We compared the baseline scenario with the reduction in influenza cases and hospitalizations achieved both per person treated and overall across all periods of implementation.

## Results

The transmission model was calibrated to the age-stratified weekly incidence data for 2014–2015 to 2018–2019 influenza seasons in four states across the USA (Fig. 1; Additional file 1: Figure S2). The model explicitly accounted for changes in transmissibility with disease progression and antiviral treatment timing. The model captured the weekly influenza trends and the age distribution of infected individuals (Fig. 1b–e; Additional file 1: Figure S2). For example, in the 2014–2015 influenza season, the calibrated model accurately showed that influenza infections peaked in week 14 in Texas, week 17 in California, week 19 in Connecticut and week 20 in Virginia (Fig. 1; Additional file 1: Figure S2). Moreover, the age distributions of symptomatic influenza cases were consistent with the empirical data in all states (Fig. 1; Additional file 1: Figure S2).

**Fig. 2:**
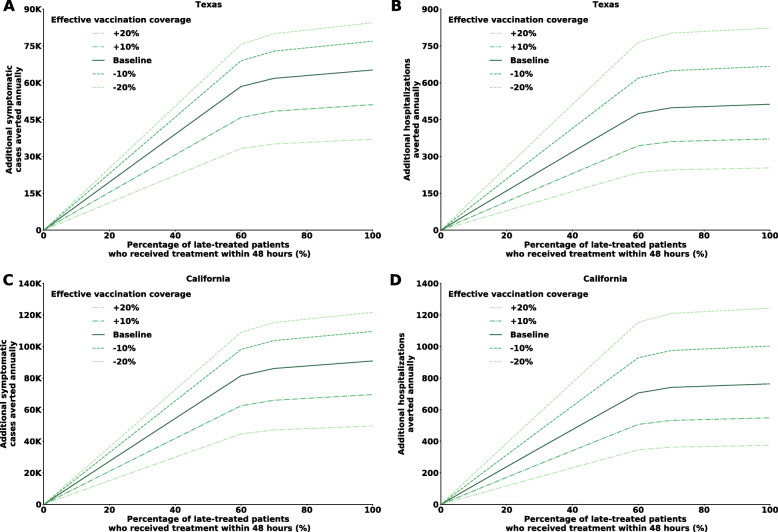
Model projection of additional symptomatic influenza cases and hospitalizations averted annually in California and Texas by increasing the number of high-risk patients who initiate treatment within 48 h of symptoms onset. Here, a proportion of high-risk patients who received antiviral treatment more than 48 h after symptom onset were assumed to receive treatment earlier. For the sensitivity analysis, we increased and decreased the effective vaccination coverage by 10% and 20%, respectively. **a**, **c** The total number of symptomatic cases averted. **b**, **d** The total number of hospitalizations averted

We simulated five influenza seasons and projected the numbers of symptomatic cases and influenza-induced hospitalizations that would have been averted by early treatment. Specifically, we evaluated the population-level benefit of increasing the proportion of high-risk individuals who initiated treatment within 48 h of symptom onset without increasing the baseline number of treated individuals (Fig. 2; Table 2; Additional file 1: Figure S3). We found that keeping the same number of treatments while providing treatment earlier could substantially decrease transmission, thereby reducing both influenza symptomatic cases and hospitalizations. If all patients were treated within 48 h of symptom onset, it would avert an additional 65,201 (36,977–84,458 range based on effective vaccination coverage) symptomatic cases and 513 (253–822) hospitalizations annually in Texas, 90,847 (49,702–121,703) cases and 764 (374–1243) hospitalizations in California, 7012 (2599–11,923) symptomatic cases and 59 (20–123) hospitalizations in Connecticut, and 18,229 (6990–28,306) symptomatic cases and 143 (49–279) hospitalizations in Virginia (Fig. 2; Table 2; Additional file 1: Figure S3). These results correspond to a 2.9% (2.1–3.7%) reduction in total symptomatic cases and a 5.5% (4.7–6.2%) reduction in total hospitalizations compared to Texas’s baseline scenario. In California, they correspond to 3.2% (2.4–3.8%) and 5.8% (5.0–6.4) reductions in total symptomatic cases and hospitalizations, respectively.

**Table 2:** Model projections of annual clinical outcomes at baseline and with different timing and coverage

Outcome	Texas		California		Connecticut		Virginia	
	Model projection (#)	Proportion^1^ (%)	Model projection (#)	Proportion^1^ (%)	Model projection (#)	Proportion^1^ (%)	Model projection (#)	Proportion^1^ (%)
**Symptomatic cases**								
Influenza symptomatic cases without antiviral treatment^2^	2,301,185 (1044395–4,135,596)		2,902,800 (1347273–5,246,107)		162,568 (52024–391,760)		460,854 (149728–1,046,816)	
Influenza symptomatic cases at baseline^2,3^	2,231,561 (1004289–4,046,681)		2,805,437 (1293191–5,117,997)		154,932 (49132–379,116)		441,098 (141980–1,016,914)	
**Symptomati**c **cases averted compared to baseline scenario**								
Treating early^4^ while keeping baseline treatment coverage^2^	65,201 (36977–84,458)	2.9 (2.1–3.7)	90,847 (49702–121,703)	3.2 (2.4–3.8)	7012 (2599–11,923)	4.5 (3.1–5.3)	18,229 (6990–28,306)	4.1 (2.8–4.9)
Treating 20% of infected individuals^2^ early^4^	116,833 (65688–152,635)	5.2 (3.8–6.5)	160,451 (87138–216,869)	5.7 (4.2–6.7)	12,543 (4611–21,568)	8.1 (5.7–9.4)	32,561 (12372–51,130)	7.4 (5.0–8.7)
**Hospitalizations**								
Hospitalizations without antiviral treatment ^2^	9896 (4365–18,385)		13,998 (6298–26,222)		902 (208–2261)		2297 (723–5546)	
Hospitalizations at baseline^2,3^	9301 (4069–17,431)		13,110 (5860–24,783)		833 (256–2119)		2130 (665–5123)	
**Hospitalizations averted compared to baseline scenario**								
Treating early^4^ while keeping baseline treatment coverage^2^	513 (253–822)	5.5 (4.7–6.2)	764 (374–1243)	5.8 (5.0–6.4)	59 (20–123)	7.1 (5.8–7.8)	143 (49–279)	6.7 (5.4–7.4)
Treating 20% of infected individuals^2^ early^4^	1021 (494–1686)	11.0 (9.7–12.1)	1506 (725–2522)	11.5 (10.2–12.4)	115 (38–247)	13.8 (11.7–14.9)	279 (95–564)	13.1 (11.0–14.2)

^1^ Percentage reduction in total symptomatic influenza cases (hospitalizations) compared to baseline scenario

^2^ Range was calculated by increasing and decreasing by 20% the state’s average effective vaccination coverage

^3^ In the baseline scenario, treatment coverage within 48 h from symptoms onset ranges between 2.7 and 4.8% (depends on age group), and after 48 from symptoms, onset ranges between 7.3 and 12.8% (depends on age group). Administering antiviral treatment within 48 h reduces transmissibility by 23.2% and risk for hospitalization by 59–79% (depends on age group)

^4^ Treating early refers to administering antiviral treatment within 48 h after symptoms onset

**Fig. 3:**
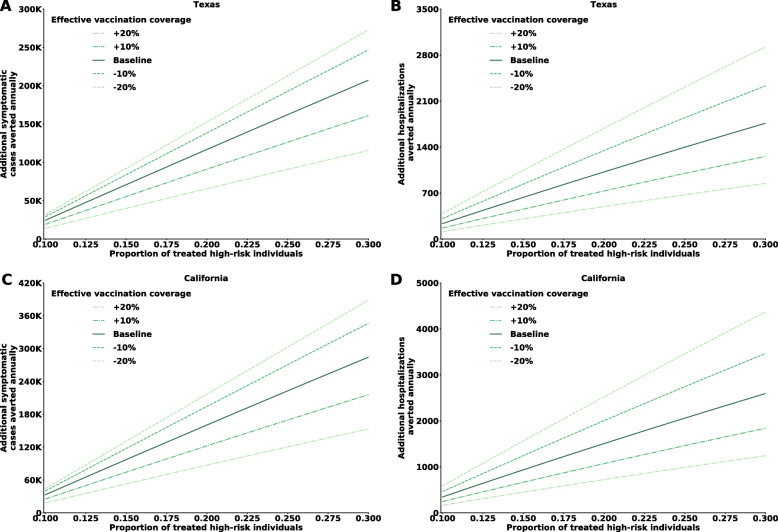
Model projections of symptomatic influenza cases and hospitalizations averted in California and Texas by increasing the portion of treated high-risk individuals who sought care and received treatment within 48 h of symptom onset. For the sensitivity analysis, we increased and decreased the effective vaccination coverage by 10% and 20%, respectively. **a**, **c** The total number of symptomatic cases averted. **b**, **d** The total number of hospitalizations averted

We also evaluated the benefit of increasing antiviral treatment coverage among high-risk individuals. Increasing early treatment coverage among individuals at high risk conferred a substantial benefit in terms of averting both cases and hospitalizations (Fig. 3; Table 2; Additional file 1: Figure S4). For example, if 20% of high-risk individuals infected with influenza were treated within 48 h of symptom onset, it would avert, on average, 116,833 symptomatic influenza cases annually in Texas, 160,451 symptomatic cases in California, 12,543 symptomatic cases in Connecticut, and 32,561 symptomatic cases in Virginia. In addition to averting symptomatic cases, this increase in treatment coverage would avert, on average, 1021 hospitalizations annually in Texas, 1506 hospitalizations in California, 115 hospitalizations in Connecticut, and 279 hospitalizations in Virginia.

Given that the benefit of treatment depends on the underlying effective vaccination coverage within the population, we also examined how variations in effective vaccination coverage would affect the benefit of treating high-risk individuals. We found that increasing effective vaccination coverage would decrease both the influenza burden and the population level benefit of treatment (Fig. 3). Nevertheless, for a 20% increase in influenza effective vaccination coverage among all age groups, our results suggest that the benefit of treatment remains substantial (Fig. 3). For example, treating 20% of infected high-risk individuals within 48 h of symptom onset would avert 65,688 symptomatic cases and 494 hospitalizations in Texas (Fig. 3a, c). In California, it would avert 87,138 symptomatic cases and 725 hospitalizations (Fig. 3b, d). In Connecticut, it would avert 4611 symptomatic cases and 38 hospitalizations, and in Virginia, it would avert 12,372 symptomatic cases and 95 hospitalizations (Table 2; Additional file 1: Figure S4).

**Fig. 4:**
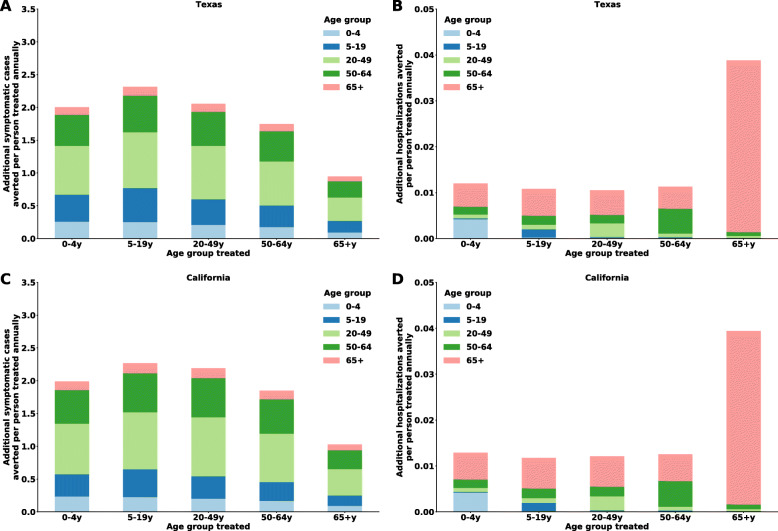
Model projections of influenza symptomatic cases and hospitalizations averted per person treated by treating each age group within 48 h of symptom onset in California and Texas. **a**, **c** Number of symptomatic cases averted per person treated for each group stratified by age. **b**, **d** Number of hospitalizations averted per person treated for each group stratified by age

To estimate the benefits of a policy that targets specific age groups for early treatment, we evaluated the effectiveness of age-targeted treatment strategies with regard to averting influenza cases and influenza-induced hospitalizations (Fig. 4; Additional file 1: Figure S5). We found that treatment of the elderly population (> 65 years old) had the highest impact on reducing hospitalizations (Fig. 4b, d). This result was driven mainly by the fact that this age group has the highest risk for influenza complications, which leads to a higher rate of hospitalization. The highest impact on reducing transmission was achieved by targeting high-risk individuals aged 5–19 years old. For example, in Texas, early treatment of the 5- to 19-year-old age group would avert 2.31 symptomatic cases per person treated, and early treatment of the > 65-year-old age group would avert 0.04 hospitalizations per person treated.

**Fig. 5:**
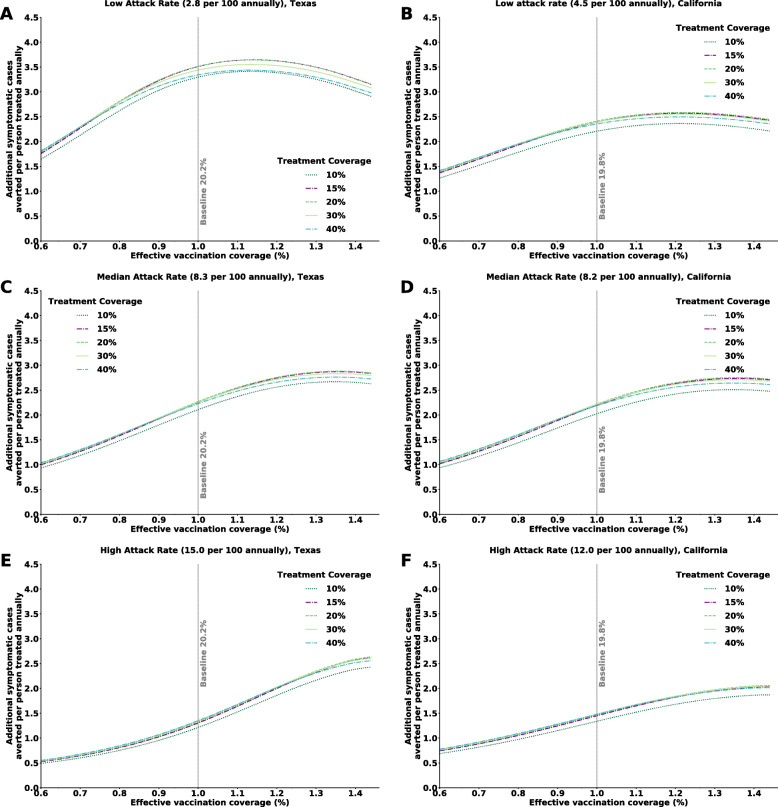
The mutual effect of attack rate and effective vaccination coverage level in California and Texas on the number of symptomatic cases averted per person treated for different treatment coverage values among high-risk individuals with influenza. Infected high-risk individuals sought care and received treatment within 48 h of symptom onset. **a**, **b** Low attack rate settings. **c**, **d** Median attack rate settings. **e**, **f** High attack rate settings

The yearly attack rate of influenza varies considerably among seasons. Thus, we explored the benefit of treating high-risk patients under different attack rates and effective vaccination coverage levels. We found that the lower the attack rate, the greater the benefit conferred per treatment (Fig. 5; Additional file 1: Figure S6). Note that in such a setting, fewer individuals will receive the treatment. Thus, the population-level effectiveness of treatment in decreasing transmission will be lower compared to settings with a higher attack rate (Fig. 3). For example, in California, in a setting of a 16 effective vaccination coverage, which is roughly 80% of the baseline effective vaccination coverage, for a high attack rate (Fig. 5f), 1.0–1.1 symptomatic cases would be averted per person treated, while for a low attack rate (Fig. 5b), 1.8–2.0 symptomatic cases would be averted per person treated in California.

In all settings considered, the benefit of treatment per person treated was found to increase with increasing treatment coverage and vaccination coverage until reaching saturation (Fig. 5). This saturation in the benefit of treatment is driven by increased herd immunity, resulting in a decrease in the indirect benefit of treatment.

There is high variability regarding the benefit of early antiviral treatment to prevent hospitalizations. Nevertheless, our sensitivity analysis (Table 3) suggested that the results remain substantial. For example, if all patients who received treatment more than 48 h after symptom onset were treated earlier, it would avert 205–718 hospitalizations in Texas, corresponding to a 2.4–8.6% reduction in total hospitalizations compared to the baseline scenario. Likewise, it would avert 311–1067 hospitalizations in California, corresponding to a 2.4–8.9% decrease compared to total hospitalization in the baseline scenario. If 20% of high-risk individuals infected with influenza were treated within 48 h of symptom onset, it would avert in Texas a rage of 397–1414 hospitalizations corresponding to a 4.5–16.8% reduction in total hospitalizations. In California, it would avert 596–2083 hospitalizations, corresponding to 4.6–17.1%, of total hospitalizations, would be averted.

**Table 3:** Sensitivity analysis of the effectiveness of early antiviral treatment with regard to averting hospitalizations

Outcome	Texas		California		Connecticut		Virginia	
	Model projection (#)	Proportion^2^ (%)	Model projection (#)	Proportion^2^ (%)	Model projection (#)	Proportion^2^ (%)	Model projection (#)	Proportion^2^ (%)
**Hospitalizations averted annually compared to baseline scenario:** ^**1**^								
Treating early^3^ while keeping baseline treatment coverage	205–718	(2.4–8.6)	311–1067	(2.4–8.9)	25–82	(3.0–10.6)	61–199	(2.9–10.1)
Treating 20% of infected individuals early^3^	397–1414	(4.5–16.8)	596–2083	(4.6–17.1)	49–157	(5.8–20.3)	116–383	(5.5–19.4)

Sensitivity analysis conducted by testing different values for (1) effectiveness of early antiviral treatment with regard to risk for hospitalizations ranges 11–89% for adults, 11–81% for children, and (2) reduction in transmissibility from individuals who have treated ranges 10.4–34.3% regardless the age group

^1^ 95% Credible Interval of total hospitalizations averted over 50,000 simulations

^2^ Percentage reduction in total hospitalizations compared to baseline scenario

^3^ Treating early refers to administering antiviral treatment within 48 h of symptoms onset

## Discussion

Our key finding shows that increasing the timeliness of the treatment of high-risk patients, even without increasing the current treatment coverage, is highly effective at reducing morbidity and mortality associated with influenza at the population level. The reason behind this finding is that the viral load of influenza is the highest during the first three days after symptom onset. Earlier treatment reduces the viral load and thus has a critical nonlinear negative effect on transmission.

Interventions that could improve the timeliness of high-risk patients seeking care and their access to timely antiviral prescriptions and thereby potentially reduce influenza-associated morbidity and mortality are urgently needed. These interventions could include education of high-risk patients, education of physicians about the benefits of providing early antiviral treatment to high-risk patients, and innovative tools to enhance the early detection of influenza infection, supporting the early initiation of treatment. These tools include providing phone consultations or remote electronic visits (virtual visits).

Vaccination remains the main tool for controlling seasonal influenza. However, vaccine efficacy varies widely between influenza seasons, and vaccination coverage remains suboptimal [7, 8]. Our study shows that the benefit of treating high-risk patients early and increasing the treatment coverage is substantial, regardless of vaccine efficacy and coverage. Counterintuitively, we found that the higher the level of effective vaccine coverage is, the higher the marginal benefit of early treatment, which is similar to the results of other studies [82, 83]. This phenomenon is driven by the fact that high effective vaccination coverage results in low disease transmission, which in turn increases the indirect benefit of treatment. This finding emphasizes the importance of antiviral treatment as a complementary effort to vaccination.

Despite the effectiveness of antivirals at reducing influenza-related morbidity and mortality, the emergence of drug resistance imposes a critical limitation on their application. Therefore, the parsimonious use of antivirals is needed to mitigate the emergence of antiviral-resistant influenza strains. Studies have suggested that to reduce the risk of antiviral overuse while maximizing their benefits to mitigate the burden of influenza, low-risk patients should be tested before treatment with antivirals, and high-risk patients with clinically diagnosed influenza infection should receive prompt treatment pending the results of a laboratory test [84]. Our study shows that increasing the timeliness of treatment without increasing the number of treated individuals would substantially increase the population-level benefit of antiviral treatment. For example, if the proportion of high-risk patients who receive treatment within the first 48 h of symptom onset increased from the baseline value of 8.1 to 14.85% (the total number of high-risk patients who receive treatment: both within and after 48 h), it could avert an additional 65,201 (36,977–84,458) symptomatic cases annually in Texas, 90,847 (49,702–121,703) symptomatic cases in California, 7012 (2599–11,923) symptomatic cases in Connecticut, and 18,229 (6990–28,306) symptomatic cases in Virginia.

The ongoing coronavirus (COVID-19) pandemic has already put unprecedented strain on the healthcare systems in many countries. As the disease continues to spread across the world, its impact on national healthcare systems has yet to be fully comprehended. In the USA, the possibility of COVID-19 transmission during the next influenza season is raising substantial concern about the healthcare system being overwhelmed by visits from high-risk patients with both COVID-19 and influenza-related complications. The specter of this challenging scenario emphasizes the importance of the results of this study and the urgent need for increased influenza vaccine coverage and timely antiviral treatment among high-risk patients in the USA.

In addition, the current COVID-19 pandemic further intensifies the importance of explicitly accounting for disease progression [85, 86]. Specifically, given that COVID-19 transmissibility peaks before symptom onset, a model that accounts for disease progression can more accurately evaluate the potential effectiveness of various interventions, including the early isolation of patients to break transmission chains. The potential impact of COVID-19 on influenza is beyond the scope of this study, and future studies should investigate the impact of COVID-19 on influenza treatment, misdiagnosis, and influenza-related complications.

Our study has several limitations that should be addressed by future studies. Although several studies have attempted to estimate the annual attack rate of influenza in the USA [1, 5, 76], the state-level rates remain unknown. Therefore, we used the national attack rate to normalize state-specific influenza cases. Moreover, we used nationwide data to estimate the treatment coverage and timeliness in each state, as state-specific data are not available. Under these assumptions, our results were qualitatively similar across all states, with quantitative differences being driven by state-specific information on population size and demographics, vaccination coverage, and influenza seasonality.

Transmission depends on the contact patterns of individuals during the period of infectiousness [34, 87]. We did not consider changes in the contact patterns due to illness. For example, when ill, one may isolate oneself from one’s surroundings and thus reduce transmission. Future models should take the effect of this change into account. To calibrate the unknown epidemiological parameters, we used the minimized squared error method. This is equivalent to maximum likelihood estimation, assuming a normal distribution of the error. However, other fitting methods can be considered by future studies [88].

## Conclusions

Increasing the timeliness and coverage of antiviral treatment among high-risk individuals has the potential to substantially reduce the burden of seasonal influenza in the USA. Timely treatment not only reduces the risk of influenza-induced hospitalization for the treated individual but may also reduce disease transmission. Even without increasing the current treatment coverage, increasing the timeliness of treatment of high-risk patients will double the effect of the current treatment coverage. Public health decision-makers should invest in continuous efforts to follow the CDC guidelines by treating influenza patients who are at high risk as soon as possible.

## Supplementary Information

**Additional file 1: **Supplementary material for: Optimizing antiviral treatment for seasonal influenza in the United States: A mathematical modeling analysis. **Table S1.** Age-specific rates *C*_*e*, *j*_ between an infected individual *e* and their contact *j*. **Figure. S1.** Daily log viral load following influenza infection for asymptomatic, symptomatic high and low-risk and treated high risk who got treated on the first three days since symptoms onset. Initiating antiviral treatment following 72 h after symptom onset: viral load will mimic symptomatic high-risk infection until three days of symptom onset. At that point, the viral load will mimic the antiviral treatment curve. **Table S2**. mean vaccination coverage [69]. **Table S3**: Fixed parameters used in the transmission model. **Table S4.** Calibrated parameters. **Figure S2. Model fit.** Time series of recorded weekly symptomatic influenza cases and model fit to Texas, California, Connecticut and Virginia (A, C, E & G). Data and model fit to the age distribution among symptomatic influenza cases (B, D, F & H). **Figure S3.** Model projection of additional symptomatic influenza cases and hospitalizations averted annually in Texas, California, Connecticut, and Virginia by increasing the number of high-risk patients who received treatment within 48 h of symptom onset. Here, a proportion of high-risk patients who received antiviral treatment more than 48 h after symptom onset was assumed to receive treatment earlier. For the sensitivity analysis, we increased and decreased the effective vaccination coverage by 10% and 20%. (A, C, E & G) Total number of symptomatic cases averted. (B, D, F & H) Total number of hospitalizations averted. **Figure S4.** Model projections of symptomatic influenza cases and hospitalizations averted in Texas, California, Connecticut, and Virginia by increasing the portion of treated high-risk individuals who sought care and received treatment within 48 h of symptom onset. For the sensitivity analysis, we increased and decreased the effective vaccination coverage by 10% and 20%. (A, C, E & G) Total number of symptomatic cases averted. (B, D, F & H) Total number of hospitalizations averted. **Figure S5.** Model projections of symptomatic influenza cases and hospitalizations averted per person treated by treating each age group within 48 h after symptom onset in Texas, California, Connecticut, and Virginia. (A, C, E & G) The number of symptomatic cases averted per person treated for each group stratified by age. (B, D, F & H) The number of hospitalizations averted per person treated for each group stratified by age. **Figure S6.** The mutual effect of attack rate and effective vaccination coverage level in Connecticut and Virginia on the number of symptomatic cases averted per person treated for different treatment coverage values among high-risk individuals with influenza. Infected high-risk individuals sought care and received treatment within 48 h after symptom onset. (A, B) Low attack rate settings. (C, D) Median attack rate settings. (E, F) High attack rate settings.

## Data Availability

The authors can confirm that all relevant data are included in the article and its supplementary information files. The medical datasets are publicly available on https://gis.cdc.gov/grasp/fluview/fluportaldashboard.html. The code supporting the simulations is publicly available on GitHub, https://github.com/matany20/Optimizing-antiviral-treatment-for-seasonal-influenza-in-the-United-States-A-mathematical-modelling.git.

## Abbreviations

ACIP: The US Advisory Committee on Immunization Practices
NAIs: Neuraminidase inhibitors
ILI: Influenza-like illness
CDC: Centers for Disease Control and Prevention
