# Supplementary material for: Optimizing antiviral treatment for seasonal influenza in the USA: a mathematical modeling analysis
Source: BMC Med. 2021 Mar 1;19:54. doi: 10.1186/s12916-021-01926-5 (PMC7917004; doi:10.1186/s12916-021-01926-5)
Supplement: Supplementary file 1 — Additional file 1: Supplementary material for: Optimizing antiviral treatment for seasonal influenza in the United States: A mathematical modeling analysis. Table S1. Age-specific rates Ce, j between an infected individual e and their contact j. Figure. S1. Daily log viral load following influenza infection for asymptomatic, symptomatic high and low-risk and treated high risk who got treated on the first three days since symptoms onset. Initiating antiviral treatment following 72 h after symptom onset: viral load will mimic symptomatic high-risk infection until three days of symptom onset. At that point, the viral load will mimic the antiviral treatment curve. Table S2. mean vaccination coverage [69]. Table S3: Fixed parameters used in the transmission model. Table S4. Calibrated parameters. Figure S2. Model fit. Time series of recorded weekly symptomatic influenza cases and model fit to Texas, California, Connecticut and Virginia (A, C, E & G). Data and model fit to the age distribution among symptomatic influenza cases (B, D, F & H). Figure S3. Model projection of additional symptomatic influenza cases and hospitalizations averted annually in Texas, California, Connecticut, and Virginia by increasing the number of high-risk patients who received treatment within 48 h of symptom onset. Here, a proportion of high-risk patients who received antiviral treatment more than 48 h after symptom onset was assumed to receive treatment earlier. For the sensitivity analysis, we increased and decreased the effective vaccination coverage by 10% and 20%. (A, C, E & G) Total number of symptomatic cases averted. (B, D, F & H) Total number of hospitalizations averted. Figure S4. Model projections of symptomatic influenza cases and hospitalizations averted in Texas, California, Connecticut, and Virginia by increasing the portion of treated high-risk individuals who sought care and received treatment within 48 h of symptom onset. For the sensitivity analysis, we increased and decrease [file 12916_2021_1926_MOESM1_ESM.docx]

Supplementary material for: Optimizing antiviral treatment for seasonal influenza in the United States: a mathematical modelling analysis

Matan Yechezkel, MSc^1*^; Martial L Ndeffo Mbah, PhD^2,3*^; Dan Yamin, PhD^1*^

^1^Department of Industrial Engineering, Tel Aviv University, 55 Haim Levanon St, Tel Aviv, Israel

^2^College of Veterinary Medicine & Biomedical Sciences and ^3^School of Public Health, Texas A&M University, College Station, Texas, 77843

^*^Authors equally contributed to the manuscript

**Table of Contents**

[1. Model overview 2](#_Toc60620861)

[2. Data set and parameters 5](#_Toc60620862)

[3. Model simulations 14](#_Toc60620863)

[4. Additional results 16](#_Toc60620864)

## Model overview

### The model

We developed a dynamic model for age-stratified influenza infection progression and transmission. Our model is a modified susceptible-infected-recovered (SIR) compartmental framework [27], whereby the population is stratified into health-related compartments, and transitions between the compartments occur over time (Main text, Fig. 1). To model age-dependent transmission, we stratified the population into age groups: 0-4 years, 5-19 years, 20-49 years, 50-64 years, and ≥ 65 years. Consistent with immunological observations [41–45], we assumed an age-dependent susceptibility reduction due to preexisting serum influenza neutralizing antibodies from previous exposure. Consistent with previous models [46–48], we assumed that individuals are fully protected for the remainder of the season upon recovery. This assumption is also supported by prospective studies demonstrating that reinfection in the same season, although possible, is rare [49, 50].

Accordingly, we stratified the population into four health-related compartments: susceptible$S_{j,k}(t)$, symptomatic infectious$I_{j,k}(t)$, asymptomatic infectious$A_{j,k}(t)$, and recovered $R_{j,k}(t)$, such that at any given time t (in days):

|  | $\sum_{j=1}^{n} \sum_{k\in\{L,H\}} \left[ S_{j,k}\left( t \right)+ I_{j,k}\left( t \right)+ A_{j,k}\left( t \right)+ R_{j,k}(t) \right]= \sum_{j=1}^{n} \sum_{k} N_{j,k} =1,$ | (1) |
| --- | --- | --- |

where the index $j\in\{1,2, . . .,n\}$ represents the age group of each individual, and the index $k\in\{L,H\}$ specifies the risk group of each individual (i.e., high-risk, or low-risk).

### Model transitioning

At the beginning of each influenza season, individuals start in the susceptible compartment $S_{j,k}(0)$. Individuals who are immune due to preexisting serum influenza neutralizing antibodies from previous exposure (proportion $\xi_{j}$ of each age group) are not included in the susceptible compartment. Thus, they transition to the recovered compartment $R_{j,k}(0)$ according to their age group and risk group. Susceptible individuals can get vaccinated based on the vaccination coverage $v_{j,k}$ and efficacy of the vaccine $\eta$. Individuals who are effectively vaccinated transition to the $R_{j,k}\left( 0 \right)$ compartment, where they are fully protected for the remainder of the season. Susceptible individuals can become infected with a force of infection $\lambda_{j}(t)$, depending on their age group $j$. Newly infected individuals can be asymptomatic with a probability of $f$ and symptomatic with a probability of $(1-f)$. Infected individuals remain in the infectious compartments $I_{j,k}\left( t \right), A_{j,k}(t)$ for $\varphi$ days and can infect their contacts based on their daily viral load and daily contact behavior (SI Appendix 2.1 Dataset and parameters). After the infectious period, individuals transition into the recovered compartment $R_{j,k}(t)$ and remain there until the end of the season. Due to lower viral load, individuals asymptomatically infected have reduced transmissibility rates than individuals symptomatically infected. Likewise, symptomatic individuals at high-risk, may have reduced transmissibility after treatment (see 2.1 Dataset and parameters).

To incorporate the evolution of infectiousness during the infection period, we explicitly track the number of symptomatic and asymptomatic infected individuals ($i_{j,k}^{\tau}, a_{j,k}^{\tau}$), respectively, with regard to their day of infection, $\tau$. Hence, $I_{j,k}(t)= \sum_{\tau=0}^{\varphi} i_{j,k}^{\tau}(t)$ and $A_{j,k}(t)= \sum_{\tau=0}^{\varphi} a_{j,k}^{\tau}(t)$. Thus, the transmission model is composed of the following system of difference equations:

|  | $S_{j,k}\left( t \right)=S_{j,k}\left( t-1 \right)-\lambda_{j}\left( t \right) \cdot S_{j,k}\left( t-1 \right),$  $R_{j,k}\left( t \right)= R_{j,k}\left( t-1 \right)+ i_{j,k}^{\tau=\varphi}\left( t-1 \right)+ a_{j,k}^{\tau=\varphi}\left( t-1 \right).$ | (2) |
| --- | --- | --- |

with daily numbers of infected individuals:

|  | $i_{j,k}^{\tau=0}\left( t \right)=\left( 1-f \right)\cdot\lambda_{j}\left( t \right) \cdot S_{j,k}\left( t-1 \right),$  $i_{j,k}^{\tau\neq0}\left( t \right)=i_{j,k}^{\tau-1}\left( t-1 \right).$  $a_{j,k}^{\tau=0}\left( t \right)=f\cdot\lambda_{j}\left( t \right) \cdot S_{j,k}\left( t-1 \right),$  $a_{j,k}^{\tau\neq0}\left( t \right)=a_{j,k}^{\tau-1}\left( t-1 \right).$  With initial conditions:  $S_{\left( j,k \right)}\left( 0 \right)= {\left( 1-\xi_{j} \right)\cdot(1-{\eta v}_{j,k} )N}_{j,k} ,$  $R_{j,k}\left( 0 \right)= {\xi_{j}\cdot N}_{j,k}+{\left( 1-\xi_{j} \right)\cdot{\eta v}_{j,k}\cdot N}_{j,k},$  $i_{j,k}^{\tau}\left( 0 \right)= a_{j,k}^{\tau}\left( 0 \right)=0.$ |  |
| --- | --- | --- |

### Force of infection

The rate at which individuals transmit influenza at time t is $\lambda_{j}(t)$. This rate depends on the combination of 1) age-specific contact rates between an infected individual and his or her contacts, 2) infectiousness of the infected individual based on his or her daily viral load, and 3) age-specific susceptibility to infection.

In the US, influenza incidence is seasonal, with a peak typically occurring in the winter, yet the driver for this seasonality remains uncertain [51]. Thus, we included general seasonal variation in the susceptibility rate of the model as

|  | $T\left( t \right)=1+\cos\left( \frac{2\pi(t-\phi)}{365} \right),$ | (3) |
| --- | --- | --- |

where $\phi$ is the seasonal offset. This formulation was previously shown to accurately capture the seasonal variation of respiratory infections in the USA [34, 52]. We incorporated age-specific contact patterns between individuals, represented by the contact rate between an infected individual in age group $e$ and each of their contacts with susceptible individuals in age group $j$, denoted by$C_{e,j}$. The contact matrix parameterization is detailed in the next section.

For high-risk individuals in age group $j$, we parameterized antiviral treatment uptake (see 2.1 Dataset and parameters). For each day during the exposed and infectious periods $(\tau)$, we incorporated the proportion of untreated or high-risk individuals denoted by $P_{j,\mathcal{T}=0}^{\tau}$, the proportion of high-risk individuals who received treatment within 48 hours since symptom onset, denoted by $P_{j,\mathcal{T}=2}^{\tau}$, and the proportion of high-risk individuals who initiated treatment within 72 hours after symptom onset, denoted by $P_{j,\mathcal{T}=3}^{\tau}$. High-risk individuals who initiated treatment more than 72 hours after symptom onset were considered to have been treated ineffectively, with treatment having no impact on disease progression or severity [53–55]. Therefore, we included them in $P_{j,\mathcal{T}=0}^{\tau}$.

Given contact with an infected host, the infectious viral load's logarithm is correlated with the transmissibility [34–37, 56]. The logarithm of the viral load depends on the risk group of the infected individual $k\in\{H,L\}$; the timing of the antiviral treatment administered to high-risk individuals $P_{j,\mathcal{T}}^{\tau};$ the day of infection $\tau$, which includes the exposed and infection periods; and the type of infection (i.e., symptomatic or asymptomatic infection) (see 2.1 Dataset and parameters). In addition, we considered the age-specific susceptibility rate of individuals, denoted by $\beta_{j}$. We calibrated the susceptibility rates to the weekly influenza records (2.2. Calibrated parameters). Taken together, the force of infection $\lambda_{j}(t)$ is given by:

|  | $\lambda_{j}\left( t \right)= \beta_{j}\cdot T\left( t \right)\cdot\left( \sum_{e=1}^{5} C_{e,j}\left( \sum_{\tau=0}^{\varphi} \sum_{\mathcal{T\in}\left\{ 0,2,3 \right\}} \log\left( VL\left( H,\tau,\mathcal{T,}symptomatic \right) \right)\cdot P_{e,\mathcal{T}}^{\tau}\cdot i_{e,H}^{\tau}\left( t-1 \right)+\sum_{\tau=0}^{\varphi} \log\left( VL\left( L,\tau,\mathcal{T}=0,symptomatic \right) \right)\cdot i_{e,L}^{\tau}\left( t-1 \right)+ \sum_{k\in\left\{ H,L \right\}} \sum_{\tau=0}^{\varphi} \log\left( VL\left( k,\tau,\mathcal{T}=0,asymptomatic \right) \right)\cdot a_{e,k}^{\tau}\left( t-1 \right) \right) \right).$ | (4) |
| --- | --- | --- |

## Data set and parameters

### Fixed parameters

*Contact mixing*

We parameterized the age-specific contact rates between an infected individual $e$ and their contact $j, C_{e,j}$ based on the contact matrix parameterized by previous studies [34, 57]. We adjusted the contact mixing matrix to our model age groups. For age groups, $5-19y$, $20-49y$ we analysed the data used to build the contact matrix using the same methods described in a previous study [34]. These contact data exhibit frequent mixing between similar age groups, moderate mixing between children and adults in their thirties (likely their parents), and infrequent mixing between other groups.

| **Table S1.** Age-specific rates$C_{e,j}$ between an infected individual $e$ and their contact $j$. |
| --- |
| 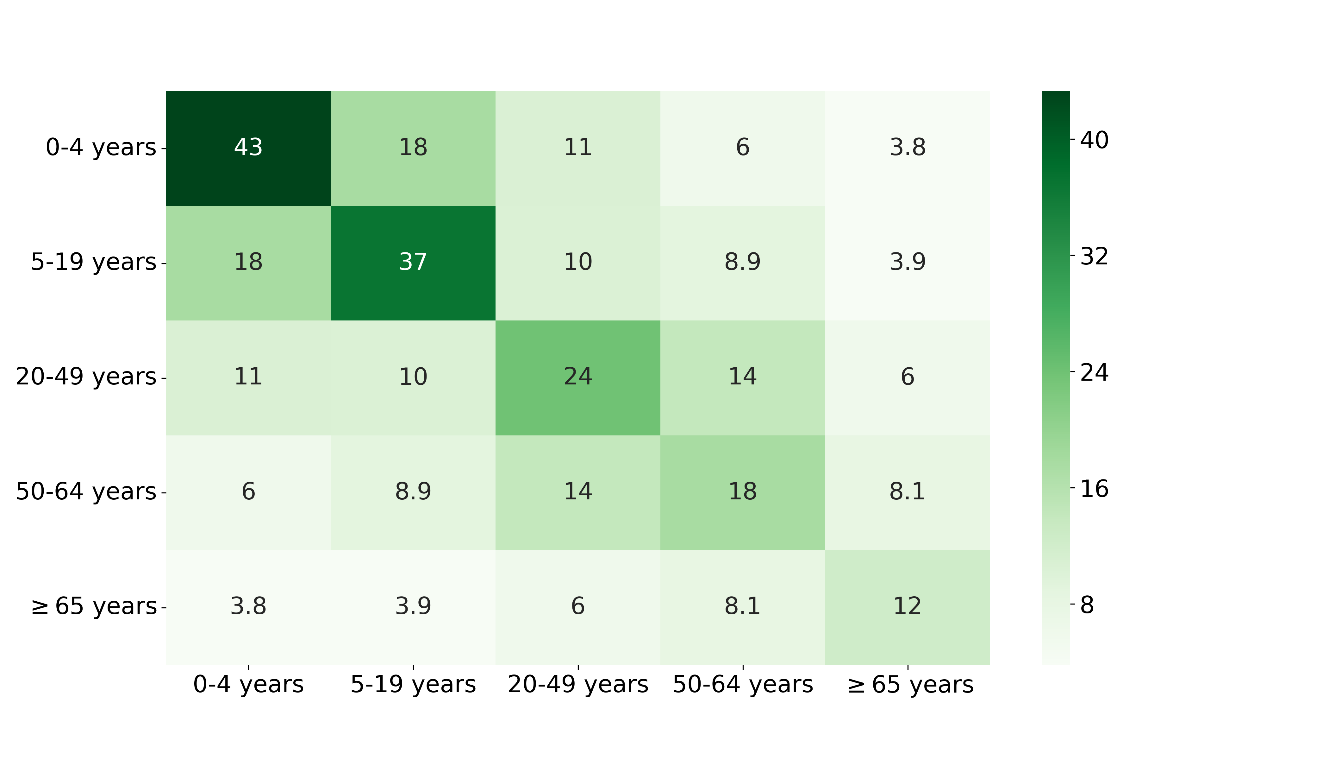 |

*Viral load*

Using recent prospective studies of the course of influenza infections in young children and adults, we estimated the viral loads in asymptomatic, symptomatic high- and low-risk and treated high-risk patients who received treatment within the first three days after symptom onset [58, 59].

The viral load in asymptomatic patients was estimated using a prospective study that tracked households in Hong Kong with patients with confirmed influenza cases [58].

For symptomatic high- and low-risk and treated high-risk patients within the first three days after symptom onset, we estimated the viral load using the data from a prospective study [59]. Concerning treated high-risk individuals, the viral load before initiating treatment was set to be the same as that for untreated individuals who were at high risk (Fig. S1).

These studies suggest that the viral load in infected individuals peaks on approximately the day of symptom onset. Moreover, untreated infected individuals at high risk have the highest level of viral shedding, while asymptomatic individuals have the lowest. Additionally, the viral shedding in individuals at high risk who are treated within 48 hours after symptom onset is reduced by 23% (CI 10-34%) compared to that in untreated individuals.

| 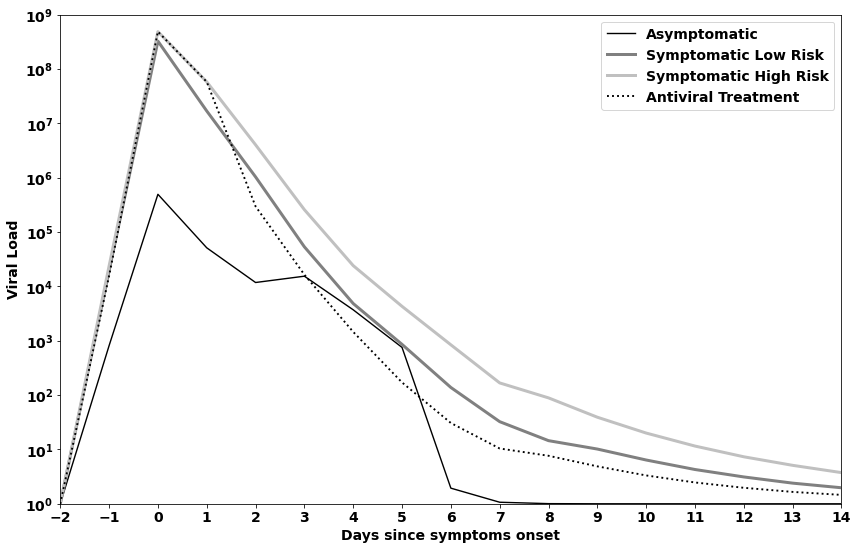 |
| --- |
| **Fig. S1.** Daily log viral load following influenza infection for asymptomatic, symptomatic high and low-risk and treated high risk who got treated on the first three days since symptoms onset. Initiating antiviral treatment following 72 hours after symptom onset: viral load will mimic symptomatic high-risk infection until three days of symptom onset. At that point, the viral load will mimic the antiviral treatment curve. |

*Hospitalizations*

To obtain the number of hospitalizations for each age and risk group, we multiplied the number of symptomatic infected individuals by the probability of hospitalization given influenza infection (see Table S3). Using data from an epidemiological study, we computed the ratio between hospitalizations and symptomatic infections stratified by age [60]. For each age group, we also estimated the risk of hospitalization for individuals at low-risk versus high-risk [61, 62]. Estimates are consistent with the US influenza hospitalization surveillance network data [63].

Infected high-risk individuals treated within three days after symptom onset have a lower probability of hospitalization [64, 65]. For individuals younger than 19 y (children), we reduced hospitalization probability given an infection by 75% [65]. For adults older than 19 y, we reduced the probability by 59% [64]. We also conducted a sensitivity analysis that considered the uncertainty of the effectiveness of treatment concerning preventing hospitalizations. We considered early treatment's effectiveness to range between 11-89% in adults and 11-81% in children [20, 64–68].

*Vaccination coverage*

We estimated the vaccination coverage based on the Centers for Disease Control and Prevention (CDC) data [69]. The data were stratified by state, age, season, and risk group. We parametrized the vaccination coverage for each season at both the national and state levels as observed in 2013-2018 (see Table S2). For national coverage, we used the median vaccination coverage in each season. As some states did not have data for the 2013-14 season, we used the average coverage of seasons 2012-13 and 2014-15 as the vaccination coverage for that season. We used the mean coverage over the five seasons as our baseline scenario. To calculate the effective vaccination coverage (see eq. (2)), the vaccination coverage $v_{j,k}$is multiplied by the vaccination efficacy $\eta$.

**Table S2**. mean vaccination coverage [69].

| **Age-group** | **0-4 years** | | **5-19 years** | | **20-49 years** | | **50-64 years** | | $\boldsymbol{\geq65}$ **years** | |
| --- | --- | --- | --- | --- | --- | --- | --- | --- | --- | --- |
| **Risk-group** | **High** | **Low** | **high** | **low** | **high** | **low** | **high** | **low** | **high** | **low** |
| **US** | 70.08% | 70.08% | 55.92% | 55.92% | 45.90% | 33.99% | 45.90% | 33.99% | 64.56% | 64.56% |
| **California** | 71.26% | 71.26% | 57.44% | 57.44% | 44.45% | 32.20% | 44.45% | 32.20% | 63.14% | 63.14% |
| **Connecticut** | 85.60% | 85.60% | 64.97% | 64.97% | 52.02% | 37.62% | 52.02% | 37.62% | 66.80% | 66.80% |
| **Virginia** | 75.58% | 75.58% | 59.10% | 59.10% | 48.54% | 39.92% | 48.54% | 39.92% | 67.72% | 67.72% |
| **Texas** | 71.60% | 71.60% | 57.87% | 57.87% | 45.38% | 31.60% | 45.38% | 31.60% | 63.10% | 63.10 |

*Treatment coverage*

Antiviral treatment is provided only to high-risk individuals who seek care in health clinics and hospitals. We assumed that following a clinic or hospital visit and treatment prescription, it would take at least one day for a patient to initiate the course of treatment. Therefore, conservatively to our findings, we assumed that the earliest effect of treatment would take place after 48 hours from symptom onset. Likewise, for people who sought care following 48-72 hours following symptoms onset, the effect of treatment was initiated following 72 hours from symptoms onset (figure S1). People who sought care following more than 72 hours from symptom onset were considered to be treated ineffectively, with treatment having no impact on disease progression and severity [53–55]. Thus, we included them with untreated individuals. Hence, the probability of infected high-risk individuals seeking care and receiving effective treatment (initiating a treatment course within 72 hours after symptom onset) is given by equation (5):

|  | $\Pr\left( getting treatment effectively \right)=\Pr\left( Seeking care\vert infected high-risk \right)\cdot\Pr\left( Seeking care\leq2 days from symptoms onset \vert Seeking care \right)\cdot\Pr\left( getting treated \vert Seeking care\leq2 days from symptoms onset care \right).$ | (5) |
| --- | --- | --- |

Estimates for these probabilities are based on large scale epidemiological studies in the US [1] [23, 24] (Table S3).

**Table S3**: Fixed parameters used in the transmission model.

| **Parameter** | **Description** | **Value** | **Justification** |
| --- | --- | --- | --- |
| $\xi_{j}$ | Proportion of individuals in age-group $j$ who are immune due to preexisting serum influenza neutralizing antibodies from previous exposure. | 0-4: 0  5-19: 0.2  20-49: 0.3  50-64: 0.25  65+: 0.25 | [41–44] |
| $N_{j,k}$ | Population size of risk group k age group $j$. | Varies between states | [1, 70] |
| $\eta$ | Mean influenza vaccination efficacy. | 0.45 | [7, 8] |
| $h_{j,k}$ | Probability of hospitalization for an infected individual in age group $j$ and risk group $k$. | k=L  0-4: 0.0006  5-19: 0.0003  20-49: 0.0006  50-64: 0.0014  65+: 0.0229  k=H  0-4: 0.0064  5-19: 0.0024  20-49: 0.0050  50-64: 0.0092  65+: 0.0680 | [61–63, 71] |
| $f$ | Probability of becoming asymptomatic given an infection. | 0.191 | [72, 73] |
| $\varphi$ | Time since exposure. | 14 days | [74] |
| $P_{j,\mathcal{T}}^{\tau}$ | Proportion of individuals at high risk from age group *J*, who receive treatment on day $\tau$from influenza exposure. Day of treatment initiation from symptom onset $\mathcal{T}\in\{\emptyset,2,3\}$ from symptom onset. $\mathcal{T=\emptyset}$ means either no treatment provided, or treatment provided late and is therefore ineffective. Early treatment is assumed to be provided to 54.5% of the total antiviral-treated patients [24]. | baseline scenario:  $\tau\geq2$  $\mathcal{T}=2 or 3$  0-4: 0.086  5-19: 0.054  20-49: 0.095  50-64: 0.074  65+: 0.063 | [1, 23, 24] |
| $C_{e,j}$ | Contact rate between an infected individual in age group $e$ and each of their contacts with susceptibility in age group $j$. |  | [34, 57] |
| VL (k,$\tau$,$\mathcal{T}$, asymptomatic/symptomatic) | Viral load of infected individuals in risk group $k$, on day of infection $\tau$ given treatment initiation day $\mathcal{T}$ and symptomatic or asymptomatic illness. |  | [58, 59] |

### Calibrated parameters

*Influenza case data and case definition*

To empirically estimate unknown epidemiological parameters, we calibrated our model to weekly cases of influenza (confirmed by viral isolation, antigen detection, or PCR) [75]. These data were collected by the National Respiratory and Enteric Virus Surveillance System of the CDC and state health departments from four different states in the United States from 2014 to 2019.

To determine the influenza cases, we multiplied the weekly number of influenza-like illness (ILI) cases by the weekly proportion of specimens positive for influenza. To account for the unreported cases in each age group, we scaled up the number of cases such that the mean attack rate between 2014-2019 matched large-scale estimates from a meta-analysis conducted in the US [5]. Although several studies have attempted to estimate state-level influenza annual attack rates in the US [1, 5, 76], the state-level rates remain unknown. Therefore, we used the national attack rate to scale up the state-specific influenza cases. Altogether, the yearly attack rate varied considerably among years and states, ranging from 2.8-15% in Texas, 4.5-12.0% in California, 5-11.7% in Connecticut, and 4.0-12.3% in Virginia. Due to the uncertainty related to influenza's actual incidence, we calibrated our model parameters for each state using different settings reflecting the lowest, average, and highest attack rates in influenza seasons.

*Calibration*

To calibrate the model to the incidence data, we minimized the squared error between the model predictions and incidence data. This is equivalent to maximum likelihood estimation, assuming a normal distribution of the error. We conducted this calibration for each of the five seasons (2014-2019) separately. For the calibration, we assumed the median US vaccination coverage by age to account for the variation in attack rates due to vaccination uptake. Additionally, we assumed the same susceptibility rate for both low- and high-risk individuals in each age group.

The final transmission model (Main text Fig. 1A) included five parameters to be estimated through model calibration: seasonal offset $\phi$; seasonal susceptibility rate $\beta_{j}$ for age group $j$: 0-4 y, 5-49 y, 50-64 y, 65+ y.

| **State** | **Season** | **Seasonal offset**  $\boldsymbol{\phi}$ | **Susceptibility among the age group 0-4 y**  $\boldsymbol{\beta}_{\boldsymbol{0-4}}$ | **Susceptibility among the age group 5-49 y**  $\boldsymbol{\beta}_{\boldsymbol{5-49}}$ | **Susceptibility among the age group 50-64 y**  $\boldsymbol{\beta}_{\boldsymbol{50-64}}$ | **Susceptibility among the age group 65+ y**  $\boldsymbol{\beta}_{\boldsymbol{65+}}$ |
| --- | --- | --- | --- | --- | --- | --- |
| **California** | **2014-15** | 68.724 | 0.0032 | 0.0015 | 0.0031 | 0.0021 |
|  | **2015-16** | 90.950 | 0.0031 | 0.0015 | 0.0030 | 0.0021 |
|  | **2016-17** | 67.791 | 0.0028 | 0.0014 | 0.0028 | 0.0020 |
|  | **2017-18** | 47.705 | 0.0031 | 0.0015 | 0.0030 | 0.0021 |
|  | **2018-19** | 92.125 | 0.0028 | 0.0015 | 0.0029 | 0.0021 |
| **Texas** | **2014-15** | 44.683 | 0.0026 | 0.0015 | 0.0029 | 0.0021 |
|  | **2015-16** | 97.567 | 0.0026 | 0.0015 | 0.0028 | 0.0022 |
|  | **2016-17** | 87.091 | 0.0028 | 0.0015 | 0.0029 | 0.0021 |
|  | **2017-18** | 73.079 | 0.0029 | 0.0016 | 0.0032 | 0.0022 |
|  | **2018-19** | 88.573 | 0.0028 | 0.0015 | 0.0031 | 0.0022 |
| **Connecticut** | **2014-15** | 82.268 | 0.0031 | 0.0015 | 0.0029 | 0.0020 |
|  | **2015-16** | 100.729 | 0.0032 | 0.0016 | 0.0029 | 0.0021 |
|  | **2016-17** | 85.539 | 0.0032 | 0.0015 | 0.0029 | 0.0020 |
|  | **2017-18** | 85.853 | 0.0036 | 0.0016 | 0.0031 | 0.0020 |
|  | **2018-19** | 80.849 | 0.0032 | 0.0016 | 0.0030 | 0.0020 |
| **Virginia** | **2014-15** | 44.862 | 0.0032 | 0.0015 | 0.0029 | 0.0020 |
|  | **2015-16** | 107.501 | 0.0031 | 0.0016 | 0.0030 | 0.0022 |
|  | **2016-17** | 95.983 | 0.0032 | 0.0016 | 0.0031 | 0.0021 |
|  | **2017-18** | 84.540 | 0.0035 | 0.0016 | 0.0032 | 0.0020 |
|  | **2018-19** | 96.904 | 0.0032 | 0.0016 | 0.0031 | 0.0021 |

**Table S4.** Calibrated parameters.

| 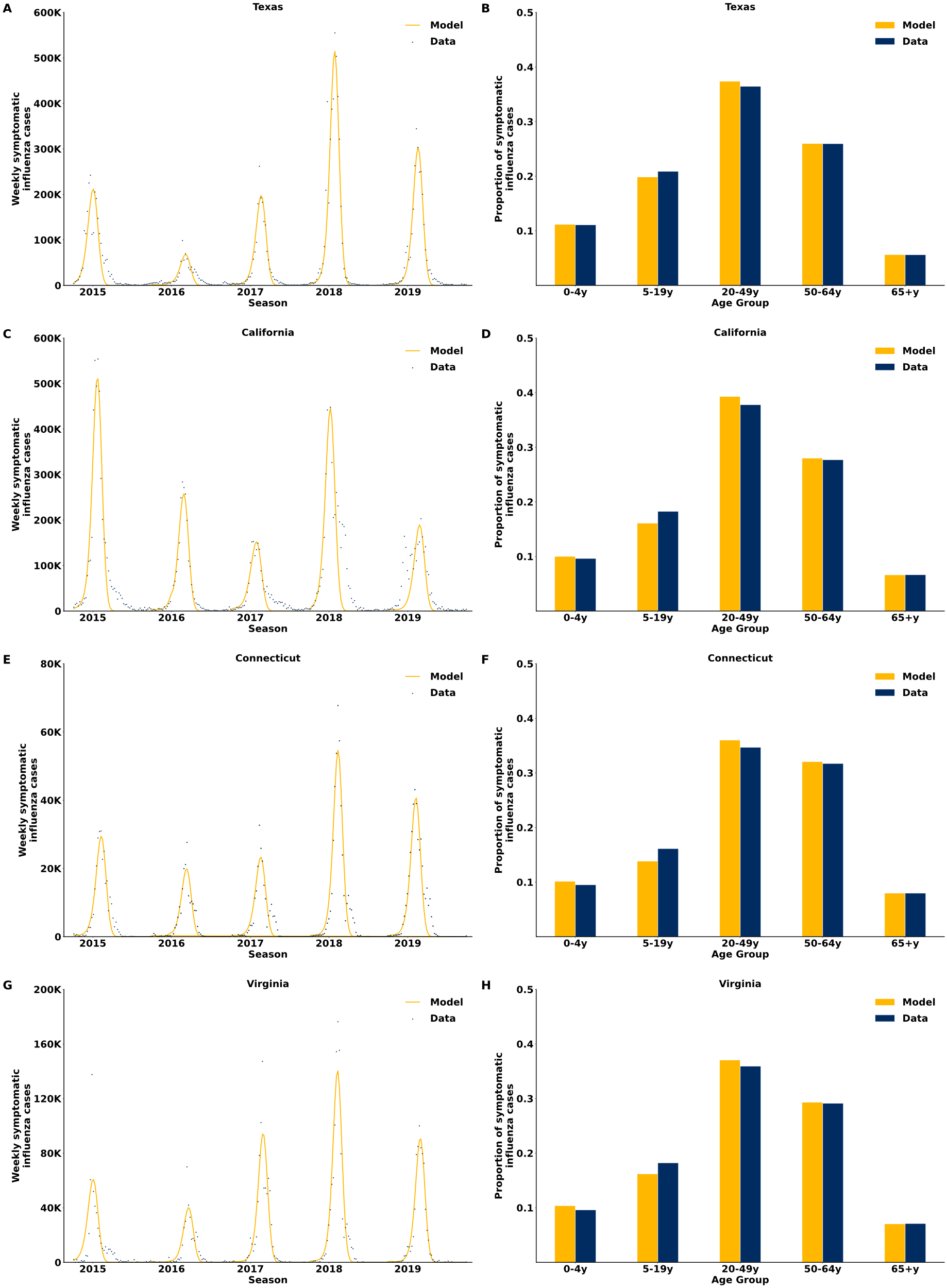 |
| --- |
| **Fig. S2. Model fit.** Time series of recorded weekly symptomatic influenza cases and model fit to Texas, California, Connecticut and Virginia (A, C, E & G). Data and model fit to the age distribution among symptomatic influenza cases (B, D, F & H). |

## Model simulations

*Main text Fig. 2*

We evaluated the additional number of cases and hospitalizations averted by early treatment (within 48 hours after symptom onset). To be conservative, we assumed treatment on the second day) in infected high-risk individuals who received treatment 48 hours after symptom onset (Main Text, Fig. 2). We ran the model for each of the five seasons. For each season, we ran the model using the average state' effective vaccination coverage level. The addition to the proportion of the infected high-risk individuals treated on the second day was done by shifting first the individuals who were treated ineffectively (more than 72 hours after symptom onset), followed by the individuals who were treated on the third day. The number of additional cases and hospitalizations averted was reported relative to the baseline scenario.

For sensitivity analyses, we examined the effect of effective vaccination coverage on the numbers of additional cases and hospitalizations averted. We increased and decreased the state's mean vaccination coverage by 10% and 20%, respectively.

*Main text Fig. 3*

We estimated the numbers of additional cases and hospitalizations averted by increasing the treatment coverage of high-risk patients, assuming that all treated patients received treatment within 48 hours after symptom onset. To assess the benefit in terms of cases and hospitalizations averted, we ran the model with treatment coverage varying from 10%-30% for each season and state using the state's average effective vaccination coverage.

For sensitivity analysis, we examined the effect of effective vaccination coverage on the number of additional cases and hospitalizations averted. We increased and decreased the state's mean vaccination coverage by 10% and 20%, respectively.

*Main text Fig. 4*

To estimate the cases and hospitalizations averted per person treated for each age group, we analyzed the scenario in which all the infected individuals at high risk in the examined age group received treatment within 48 hours after symptom onset. For the rest of the age groups, we set the timing of the initiation of treatment to 48 hours after symptom onset without changing the baseline treatment coverage.

*Main text Fig. 5*

We conducted two-way sensitivity analyses to investigate the mutual impact of seasonal attack rate and vaccination coverage in each state on the number of cases averted per person treated. We ran the model with varying proportions of infected high-risk individuals who sought care and were treated within 48 hours after symptom onset from 10%-30% while increasing and decreasing the state's average effective vaccination coverage by 10%-40%. We analyzed median, high, and low seasonal attack rate settings in each state. The high and low attack rates were informed by the seasons with the lowest and highest attack rates in each state between 2014 and 2019.

*Main text Table 3 – Hospitalization sensitivity analysis*

We conducted a sensitivity analysis to examine the effectiveness of early antiviral treatment with regard to hospitalizations. We ran 50,000 simulations for each state. In each simulation, we decreased and increased the effect of early antiviral treatment with regard to both reducing transmissibility and reducing hospitalizations based on the sampled percentile. The reduction in hospitalizations for adults >19 y ranged from 11-89%, and for individuals $\leq$19 y, it ranged from 11-81%. The reduction in transmissibility ranged from 10.4-34.3% [20, 37, 58, 64–68].

## Additional results

| 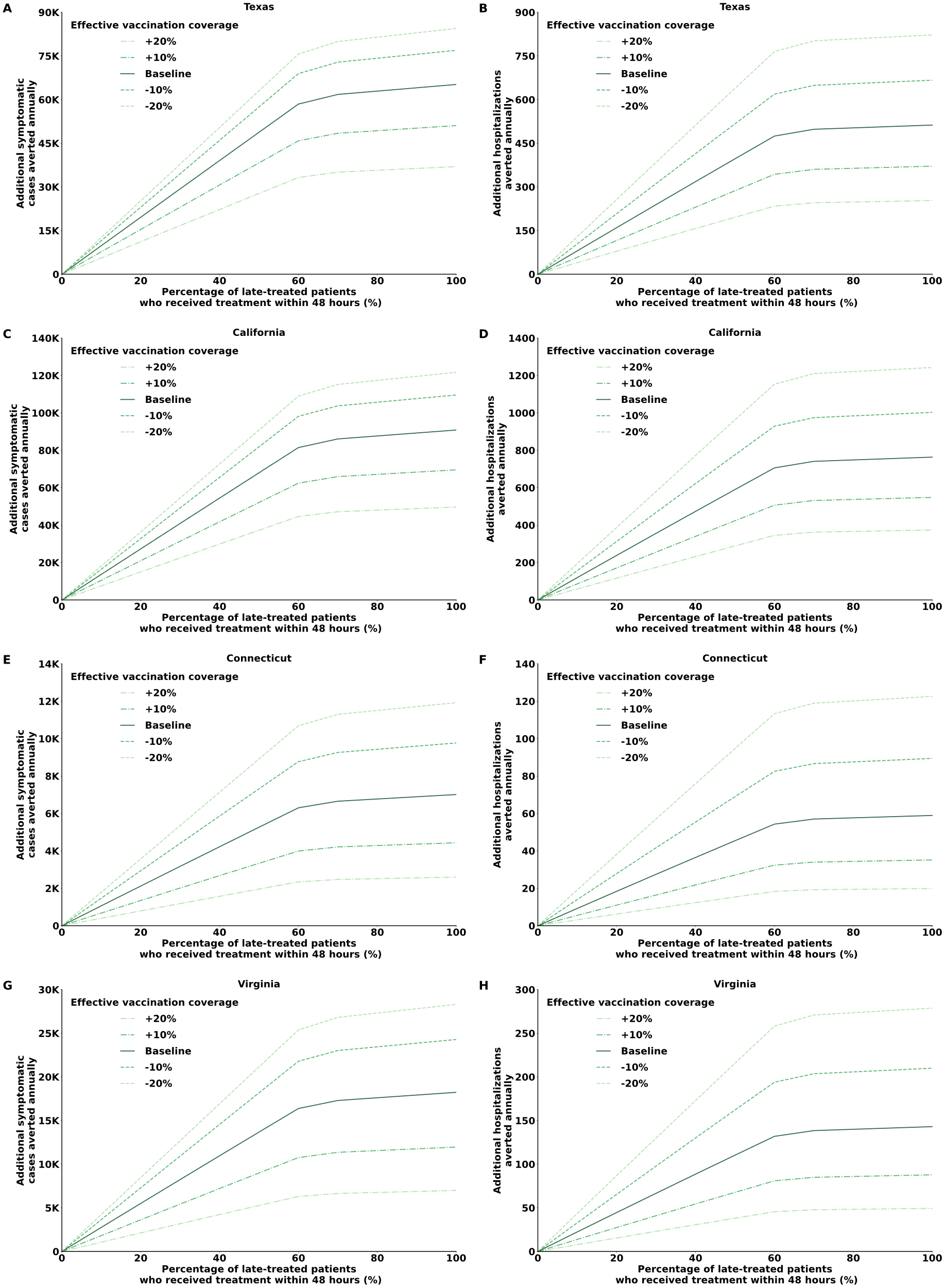 |
| --- |
| **Fig. S3.** Model projection of additional symptomatic influenza cases and hospitalizations averted annually in Texas, California, Connecticut, and Virginia by increasing the number of high-risk patients who received treatment within 48 hours of symptom onset. Here, a proportion of high-risk patients who received antiviral treatment more than 48 hours after symptom onset was assumed to receive treatment earlier. For the sensitivity analysis, we increased and decreased the effective vaccination coverage by 10% and 20%. (A, C, E & G) Total number of symptomatic cases averted. (B, D, F & H) Total number of hospitalizations averted. |


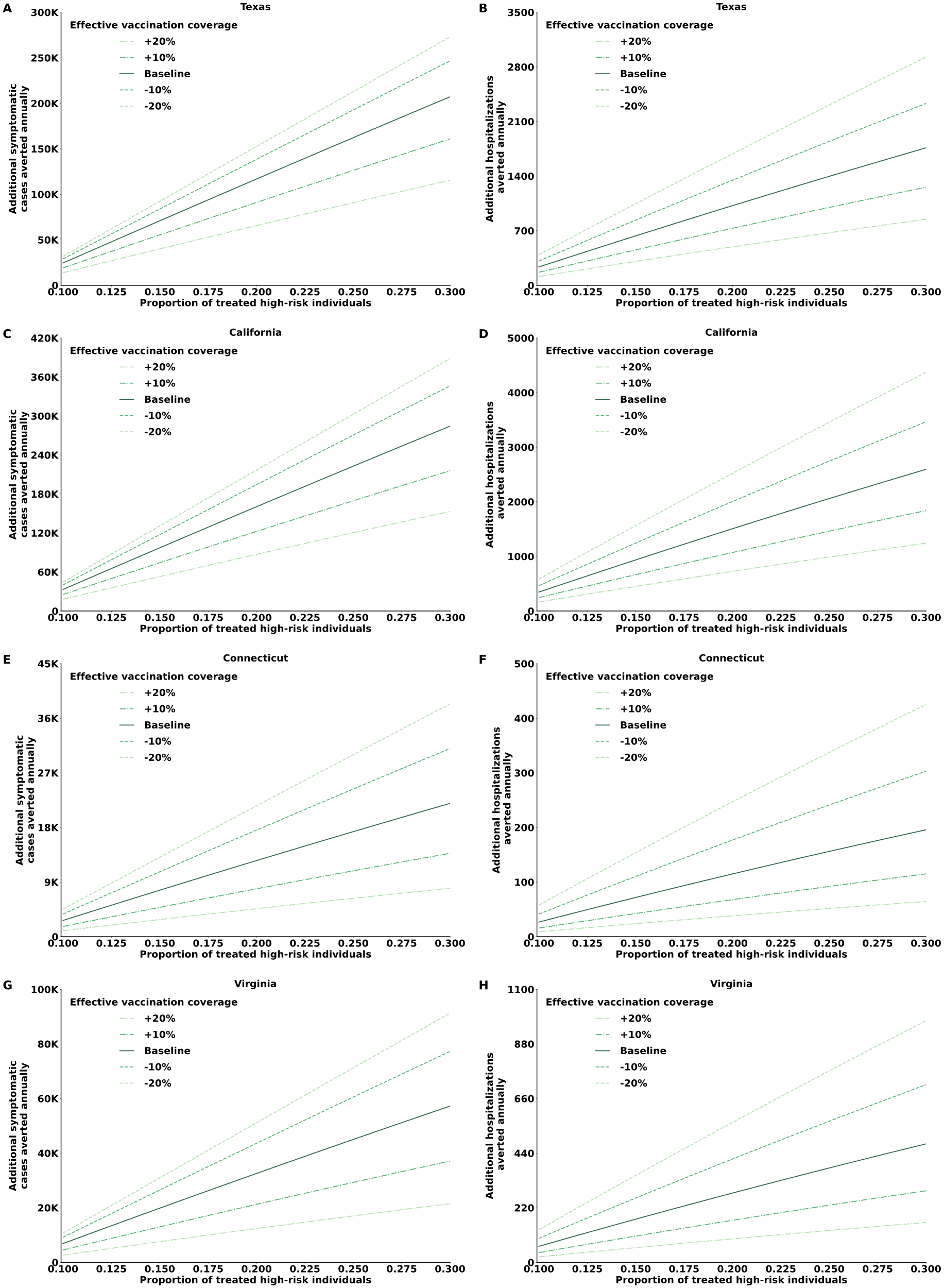


| **Fig. S4.** Model projections of symptomatic influenza cases and hospitalizations averted in Texas, California, Connecticut, and Virginia by increasing the portion of treated high-risk individuals who sought care and received treatment within 48 hours of symptom onset. For the sensitivity analysis, we increased and decreased the effective vaccination coverage by 10% and 20%. (A, C, E & G) Total number of symptomatic cases averted. (B, D, F & H) Total number of hospitalizations averted. |
| --- |

| 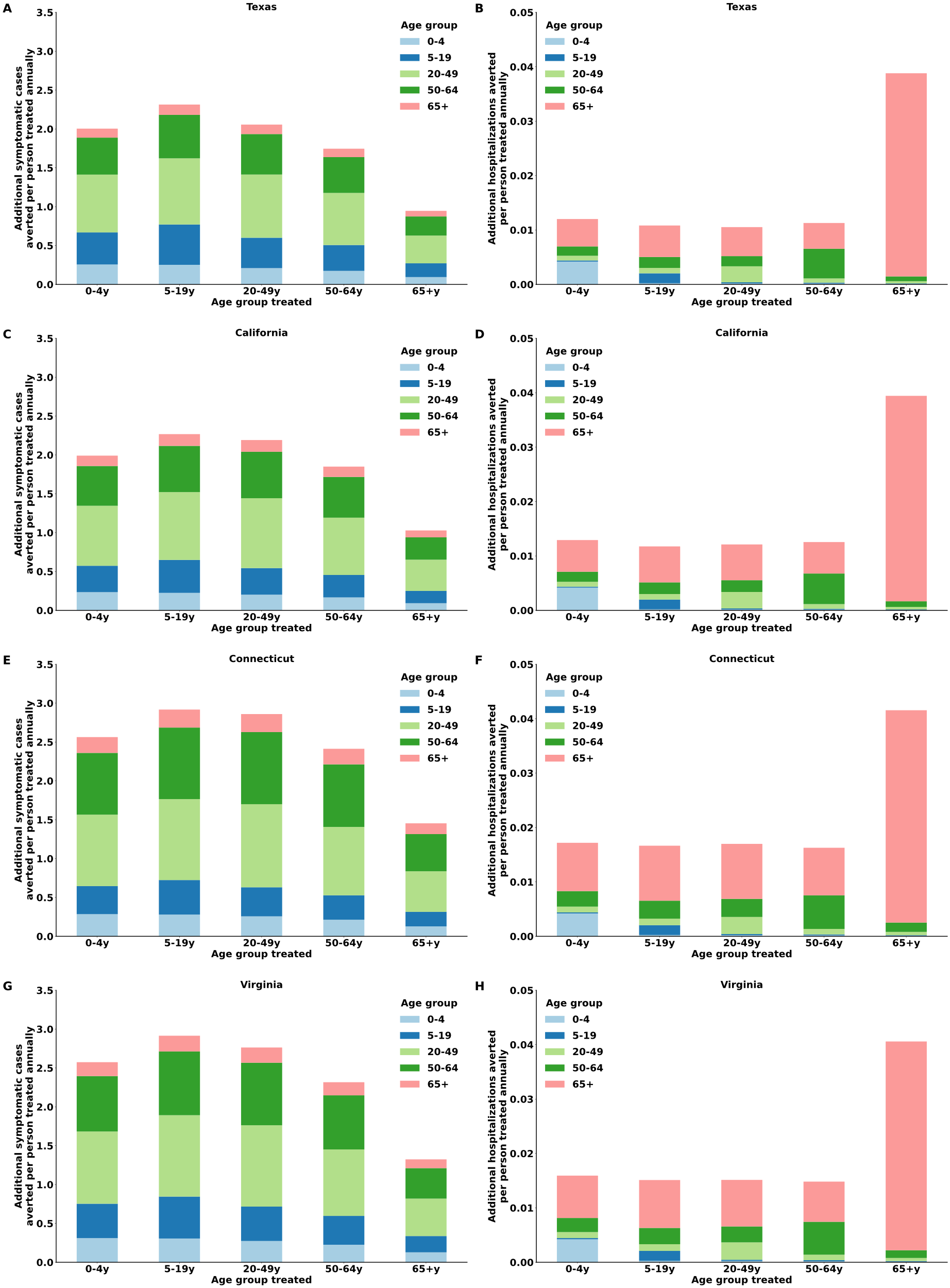 |
| --- |
| **Fig. S5.** Model projections of symptomatic influenza cases and hospitalizations averted per person treated by treating each age group within 48 hours after symptom onset in Texas, California, Connecticut, and Virginia. (A, C, E & G) The number of symptomatic cases averted per person treated for each group stratified by age. (B, D, F & H) The number of hospitalizations averted per person treated for each group stratified by age. |

| 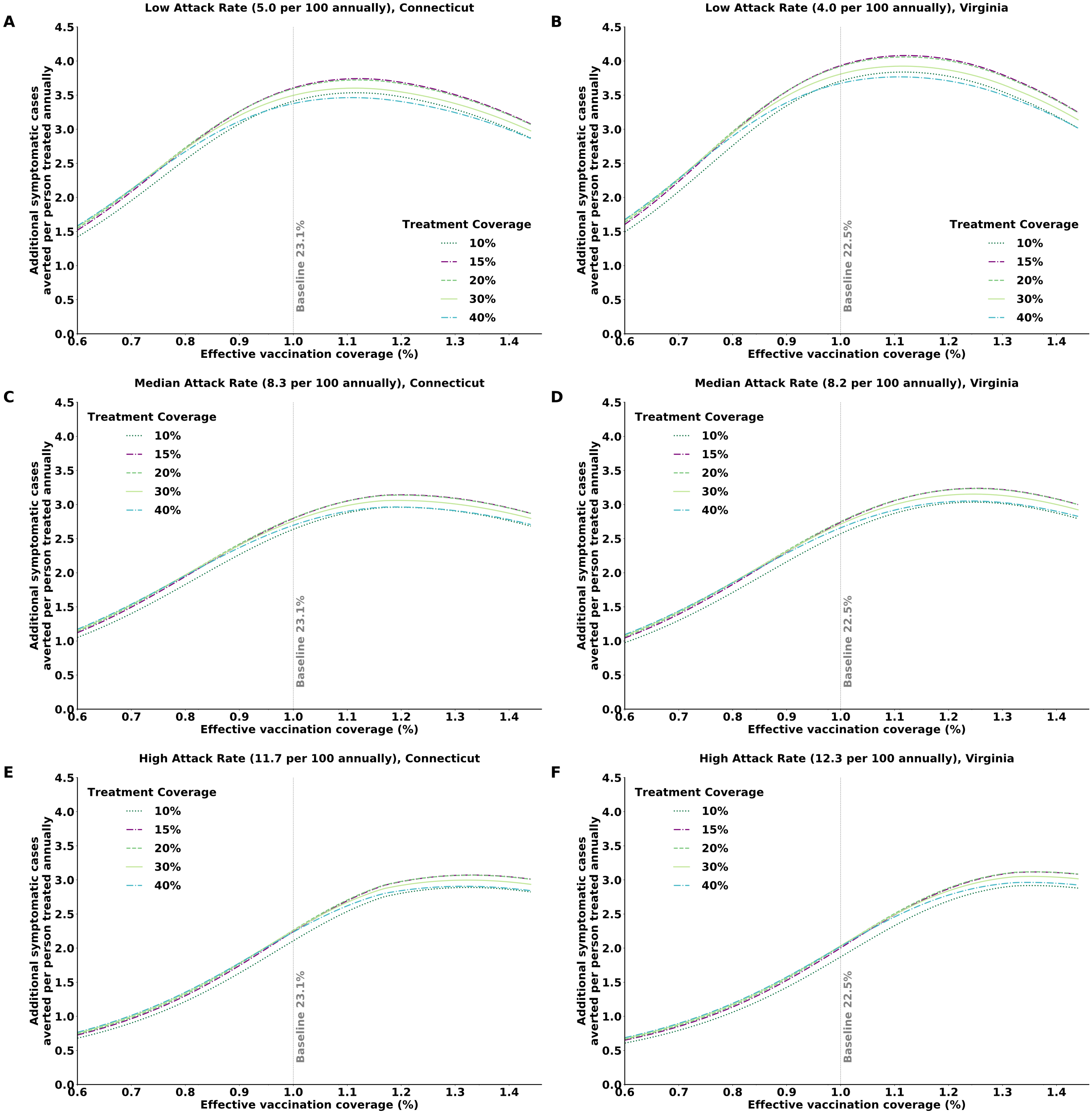 |
| --- |
| **Fig. S6.** The mutual effect of attack rate and effective vaccination coverage level in Connecticut and Virginia on the number of symptomatic cases averted per person treated for different treatment coverage values among high-risk individuals with influenza. Infected high-risk individuals sought care and received treatment within 48 hours after symptom onset. (A, B) Low attack rate settings. (C, D) Meidan attack rate settings. (E, F) High attack rate settings. |
